# High frequency oscillations in epileptic and non-epileptic human hippocampus during a cognitive task

**DOI:** 10.1038/s41598-020-74306-3

**Published:** 2020-10-23

**Authors:** Martin Pail, Jan Cimbálník, Robert Roman, Pavel Daniel, Daniel J. Shaw, Jan Chrastina, Milan Brázdil

**Affiliations:** 1 First Department of Neurology, Brno Epilepsy Center (Full member of the ERN EpiCARE), St. Anne’s University Hospital and Medical Faculty of Masaryk University, Pekařská 53, Brno, 65691 Czech Republic; 2 International Clinical Research Center, St. Anne’s University Hospital, Brno, Czech Republic; 3 CEITEC – Central European Institute of Technology, Masaryk University, Brno, Czech Republic; 4 School of Life and Health Sciences, Aston University, Birmingham, UK; 5 Department of Neurosurgery, Brno Epilepsy Center, St. Anne’s University Hospital and Medical Faculty of Masaryk University, Brno, Czech Republic

**Keywords:** Epilepsy, Hippocampus

## Abstract

Hippocampal high-frequency electrographic activity (HFOs) represents one of the major discoveries not only in epilepsy research but also in cognitive science over the past few decades. A fundamental challenge, however, has been the fact that physiological HFOs associated with normal brain function overlap in frequency with pathological HFOs. We investigated the impact of a cognitive task on HFOs with the aim of improving differentiation between epileptic and non-epileptic hippocampi in humans. Hippocampal activity was recorded with depth electrodes in 15 patients with focal epilepsy during a resting period and subsequently during a cognitive task. HFOs in ripple and fast ripple frequency ranges were evaluated in both conditions, and their rate, spectral entropy, relative amplitude and duration were compared in epileptic and non-epileptic hippocampi. The similarity of HFOs properties recorded at rest in epileptic and non-epileptic hippocampi suggests that they cannot be used alone to distinguish between hippocampi. However, both ripples and fast ripples were observed with higher rates, higher relative amplitudes and longer durations at rest as well as during a cognitive task in epileptic compared with non-epileptic hippocampi. Moreover, during a cognitive task, significant reductions of HFOs rates were found in epileptic hippocampi. These reductions were not observed in non-epileptic hippocampi. Our results indicate that although both hippocampi generate HFOs with similar features that probably reflect non-pathological phenomena, it is possible to differentiate between epileptic and non-epileptic hippocampi using a simple odd-ball task.

## Introduction

The discovery of high-frequency electrographic activity represents one of the essential milestones not only in epilepsy research, but also in cognitive science over the past few decades. These transient high and very high-frequency oscillations (HFOs/VHFOs) in invasive EEG (stereoelectroencephalography; SEEG) have been recorded repeatedly in several allocortical and neocortical structures. These short-lasting field potentials, both ictal and interictal phenomena, can be divided further into “ripples” (80–250 Hz), “fast ripples” (250–600 Hz), “very fast ripples” (VFR; 600–1000 Hz), and “ultra-fast ripples” (UFR; 1–2 kHz), all of which have been studied widely in humans under physiological and pathological conditions^1–12^.

HFOs are believed to stem from the short-term synchronization of neuronal populations and their activity, and it appears that they are connected to normal as well as pathological brain functions^6,13^. While physiological HFOs seem to represent summated synchronous inhibitory postsynaptic potentials (IPSP) generated by interneuronal cell subpopulations regulating the principal cell activity and their discharges^14^. Epileptic HFOs might reflect the field potentials which are formed by the activity from clusters of abnormal synchronously bursting pyramidal cells, generating population spikes, and decreased inhibitory interneuron firing^6,15^. The detected HFO frequency can be determined purely from the behavior and activity of one cell subpopulation (“pure” HFOs). However, the observed HFOs (especially beyond the physiologic limits of neuronal firing; > 300 Hz), may also represent the net frequency of neuronal populations, more specifically due to the activity of different cell subpopulations of synchronized neurons between which is a phase delay in activity. Each cell assembly then usually oscillate with a lower frequency than observed (“emergent” HFOs)^16,17^.

There is evidence that ripple generation is influenced by larger networks; smaller networks are involved in fast ripples and even less in VFR and UFR generation, which can be observed focally^9^. Interest in HFOs is related primarily to the localization of the epileptogenic zone, since they are considered as being more focal and specific than classical epileptic spikes^18^ that are only partially concordant with the epileptogenic zone^19^. As HFO rates are higher in focal seizure-generating tissue, they have attracted attention as a possible clinical biomarker^3^.

Unfortunately, pathological and physiological HFOs cannot be distinguished by rate of their occurrence, as some regions are identified as generators of physiological HFOs^12^. Moreover, another significant problem, has been the fact that pathological HFOs overlap in frequency with physiological HFOs associated with normal brain function^8,20–22^. How these two types of electrographic phenomena can be separated remains unclear^6^, as frequency and/or amplitude analysis alone seem to be insufficient for their delineation^23–25^. Therefore, the translation of HFOs into clinical practice is hindered by the inability to differentiate between pathological and normal HFOs in SEEG recordings. And so, more than twenty years after their discovery, there still exist questions about HFOs as biomarkers of epileptogenic brains and epileptogenic zones, and about their utility in clinical practice^12,26,27^.

Until now, various analytical approaches have been sought to distinguish pathological and physiological HFOs. The methods used most commonly are based on, for example, a specific regional distribution in mesial temporal structures^28^; the association of HFOs with epileptiform discharges^4,26,29^, slow waves^30^ o spindles^31^, or the difference between HFOs produced spontaneously and those induced by a cognitive task^8,23,24^. In the recent study of Sakuraba et al., epileptogenic region was determined based on less suppressive effect of REM sleep on HFOs in contrast to non-epileptogenic/physiological region^32^. Some authors have suggested separating HFOs by clustering them based on features such as frequency, duration, and amplitude^24,25,33^. Other reports have addressed this problem proposing several methods for dissociating different origins based on the width of the spectral frequency content of individual events, number of distinct cycles observed, and the presence of actual oscillations in the unfiltered raw signal^34,35^. Finally, when investigating electrophysiological brain recordings, the term HFO should be used to describe true high-frequency local field potential oscillations in the invasive EEG, that is oscillations visible in the raw recording and not the high-frequency Fourier components from a bandpass filter^36^. The ability to distinguish between pathological and physiological HFOs is crucial for understanding normal cognitive functions and no less important for the translation of HFOs into clinical practice.

In a recent study, we tested the hypothesis whether the presumed effect of cognitive task on hippocampal ripples can be used as a new approach for distinguishing pathological HFOs in the epileptic hippocampus (EH) from physiological HFOs in the non-epileptic hippocampus (NEH)^8^. To differentiate them, a simple oddball task was used. This study revealed different and, in some cases, opposing behavior of ripples within EH and NEH: Ripples were significantly more reduced during a cognitive task than in a resting period in EH, but in NEH this difference remained statistically marginal^8^. Moreover, we observed a significant suppression of ripple rate in the first second after stimulus onset only in NEH^8^. Importantly, however, we did not examine fast ripples due to a low sampling frequency.

In the present study, we tested the hypothesis that not only ripples, but also fast ripples are modulated by cognitive tasks. We aimed to find a distinct impact of a cognitive task on HFOs (the rate and other HFO characteristics) within EH and NEH. To test the hypothesis, we analyzed hippocampal SEEG of 15 patients during resting period and during a simple cognitive oddball task.

## Methods

### Subjects

In our study we included 15 patients (7 females) ranging in age from 24 to 56 (mean: 38.3 ± 9.3) years. All patients suffered from medically intractable focal temporal epilepsies. For demographic and clinical characteristics of the included subjects, see Table 1. In most patients, chronic anticonvulsant medication was reduced slightly for the purposes of video-SEEG monitoring. The study procedures were approved by Masaryk University and St. Anne’s University Hospital Ethics Committees. All subjects gave their written informed consent prior to the study investigation. All methods were performed in accordance with the relevant guidelines and regulations.

**Table 1:** Demographic and clinical data.

Subject	Gender	Age at SEEG	FS	Age at Seizure onset	MRI before SEEG	Side of epilepsy	SOZ	Intervention/ histopathology	Postoperative outcome Engel (follow-up, year)	Number of analyzed contacts in EH	Number of analyzed contacts in NEH	Number of analyzed events in EH (spikes/R/FR)	Number of analyzed events in NEH (spikes/R/FR)
1	F	26	–	17	Normal	Left	Left hippocampus	Left AMTR/ FCD IB	IA (5)	6 (left)	3 (right)	1950/517/835	205/69/302
2	F	56	–	28	Right hippocampal atrophy	Right	Right hippocampus	Right AMTR/not available	IIIA (5)	6 (right)	–	1623/1991/1380	–
3	M	40	–	1	Left hippocampal atrophy	Left	Left hippocampus	Left AMTR/negat	IA (5)	8 (left)	–	4501/2212/1454	–
4	M	38	–	27	Normal	Bilaterally	Hippocampus bilaterally (mainly right side)	VNS	–	3 (right)	–	812/420/404	–
5	M	41	–	33	Focal hyperintensity within right basal temporal lobe	Right	Right hippocampus, lesion	Right AMTR/FCD IIIb ganglioglioma	IA (5)	7 (right)	3 (left)	1735/672/623	499/209/286
6	F	33	–	2	Postencephalitic changes of left T lobe, left hippocampal atrophy	Left	Left hippocampus, lesion	Left AMTR/hippocampal sclerosis, postencephalitic changes	IA (5)	6 (left)	5 (right)	1241/1029/97	155/50/297
7	M	35	–	21	Bilateral hippocampal atrophy, RX > LT	Bilaterally	Hippocampus bilaterally (mainly right side)	VNS	–	7 (right)	–	2314/978/560	–
8	M	37	FS	31	Normal	Bilaterally	Hippocampus bilaterally (mainly left side)	Left AMTR/negat	IA (4)	8 (left)	–	1149/1247/1002	–
9	F	27	–	9	Left hippocampal atrophy	Left	Left hippocampus	Left AMTR/FCD IIIA	IIIA (4)	7 (left)	–	2767/1878/1767	–
10	M	51	FS	2	Right hippocampal atrophy	Right	Right hippocampus	Right AMTR/hippocampal sclerosis	IA (4)	5 (right)	7 (left)	636/568/895	222/171/435
11	M	24	–	10	Left hippocampal atrophy, mild posttraumatic gliosis of left pericentral region	Left	Left hippocampus	Left AMTR/hippocampal sclerosis	IIA (4)	5 (left)	–	815/507/228	–
12	F	33	–	29	Asymetry of colateral sulci in temporal lobe	Left	Temporal pole and lateral temporal cortex	Resection of temporal pole and anterior part of lateral temporal cortex/negat	IIIA (4)	–	5 (left)	–	308/69/125
13	F	45	–	26	Left hippocampal atrophy	Left	Left T pole	Left AMTR / negat	IIIA (4)	–	6 (right)	–	155/53/328
14	F	36	–	16	Nodular heterotopia along dorsal part of lateral ventricle and lateral cortex TO left	Left	Left lateral cortex TO junction	Lateral cortical resection TO left/FCD IIA	IIA (4)	–	4 (left)	–	326/181/137
15	M	53	–	33	Left hippocampal atrophy	Left	Left hippocampus	Left AMTR	IA (1)	8 (left)	–	3560/1641/1064	–

M = male, F = female; SEEG = stereoelectroencephalography; T = temporal; O = occipital; FS = febrile seizures; SOZ = seizure onset zone; AMTR = anteromedial temporal resection; FCD = focal cortical dysplasia; EH = epileptic hippocampus; NEH = non-epileptic hippocampus; VNS = vagus nerve stimulation, R = ripples, FR = fast ripples.

### EEG recordings

Patients underwent the implantation of depth electrodes as part of their evaluation for pharmacoresistant focal epilepsy in order to localize seizure origin prior to surgical treatment. The location of the implanted electrodes was determined by clinical requirements. Each patient received 3–14 intracerebral electrodes containing either 5, 8, 10 or 15 individual contacts, in the temporal lobe and facultatively in other brain lobes using the Talairach stereotaxic system^37^. Standard platinum depth electrodes (ALCIS) were used (diameter = 0.8 mm; inter-contact distance = 1.5 mm, contact surface area = 5 mm^2^; contact length = 2 mm). After implantation, each patient underwent MRI scanning to localize electrode placement. We used a 192-channel research EEG acquisition system (M&I; Brainscope, Czech Republic) for recording 30 min of an awake resting interictal period as well as the cognitive task. The sampling rate was 25 kHz and dynamic range of ± 25 mV with 10 nV (24 bits). The EEGs were low-pass filtered and downsampled to 5 kHz for further processing. All recordings were referenced to the average of intracranial signals. EEG data from a total of 111 electrode contacts positioned in either epileptic hippocampi (76) or non-epileptic hippocampi (35) were investigated (Table 1). No other structures were analyzed for the presence of spikes and HFOs. In identifying epileptic (EH) and non-epileptic hippocampi (NEH), we followed a process similar to that reported elsewhere^8,24^—specifically, based on the results of a standard visual analysis of interictal and ictal SEEG recordings: EH were identified by the presence of a seizure onset zone (confirmed by recording multiple seizures): the site in which contacts showed the first EEG ictal activity, with characteristic desynchronization and low voltage fast activity pattern. As presented in the Table 1, epileptic hippocampus was selected in 12 patients. A unilateral hippocampal epileptic region was found in 9 patients (6 patients with Engel IA (seizure free), 1 patient with Engel IIA (histologically confirmed hippocampal sclerosis), 2 patients with Engel IIIA (in one case histologically confirmed hippocampal sclerosis)). Bilateral epileptic hippocampal regions were determined in three, in which we analyzed the data only within a more pathologically active hippocampus. Putative NEH were defined by the absence of (a) a seizure onset zone and (b) frequent interictal spikes (IEDs; > 50 per 10 min). The putative non-epileptic hippocampi with spiking above the threshold were visually reviewed whether the IEDs were propagated from other brain structures. The putative non-epileptic hippocampi that generated IEDs were excluded from the analysis. NEH were identified in either the left or right hippocampus in extramesiotemporal epilepsy, but contralateral to the epileptogenic hippocampus in unilateral mesiotemporal epilepsy. In this way, each hippocampus could be classified either as epileptic or non-epileptic. We always analyzed all the electrode contacts within a particular hippocampus. In each subject, all the obtained data were reviewed to identify artifactual and pathological traces by expert neurologists (M.B. and M.P.).

Awake resting state was recorded with the subject’s eyes closed with the minimization of possible external stimuli.

### Behavioral tasks

Subjects were seated comfortably in a moderately lighted room. A monitor screen was placed approximately 100 cm in front of their eyes. During the task, they were asked to focus their gaze on a small fixation point in the center of the monitor screen. We performed a standard visual oddball task: three types of stimuli (target, frequent, and distractor) were presented in the center of the screen (black background) for 500 ms in random order at a ratio of 1:4.6:1. The interstimulus interval varied randomly between 4 and 6 s. Specifically, the experimental stimuli comprised clearly visible yellow capital letters “X” (target), “O” (frequent), and various other capital letters (distractor). The number of targets was 50. The task was divided into four blocks, each block consisting of 12 or 13 target stimuli. Each subject was instructed to count the target stimuli sub-vocally and to report the calculated number after each block.

### Data analysis

Using a modified pipeline for automated HFO detection^21^, we analyzed potential ripple and fast ripple rates in EH and NEH. We also carried out an automated detection of interictal spikes in the dataset used in this study^38^. Further, we compared the influence of the cognitive task on HFO and spikes occurrence in EH and NEH. Analyses of the HFO and spike occurrences and HFO features (relative amplitude, duration, and spectral entropy) were performed separately in the first 10-min window of the visual oddball task (i.e. throughout the whole epoch, not just in the short segments after specific stimuli) and during the resting state.

**Figure 1:**
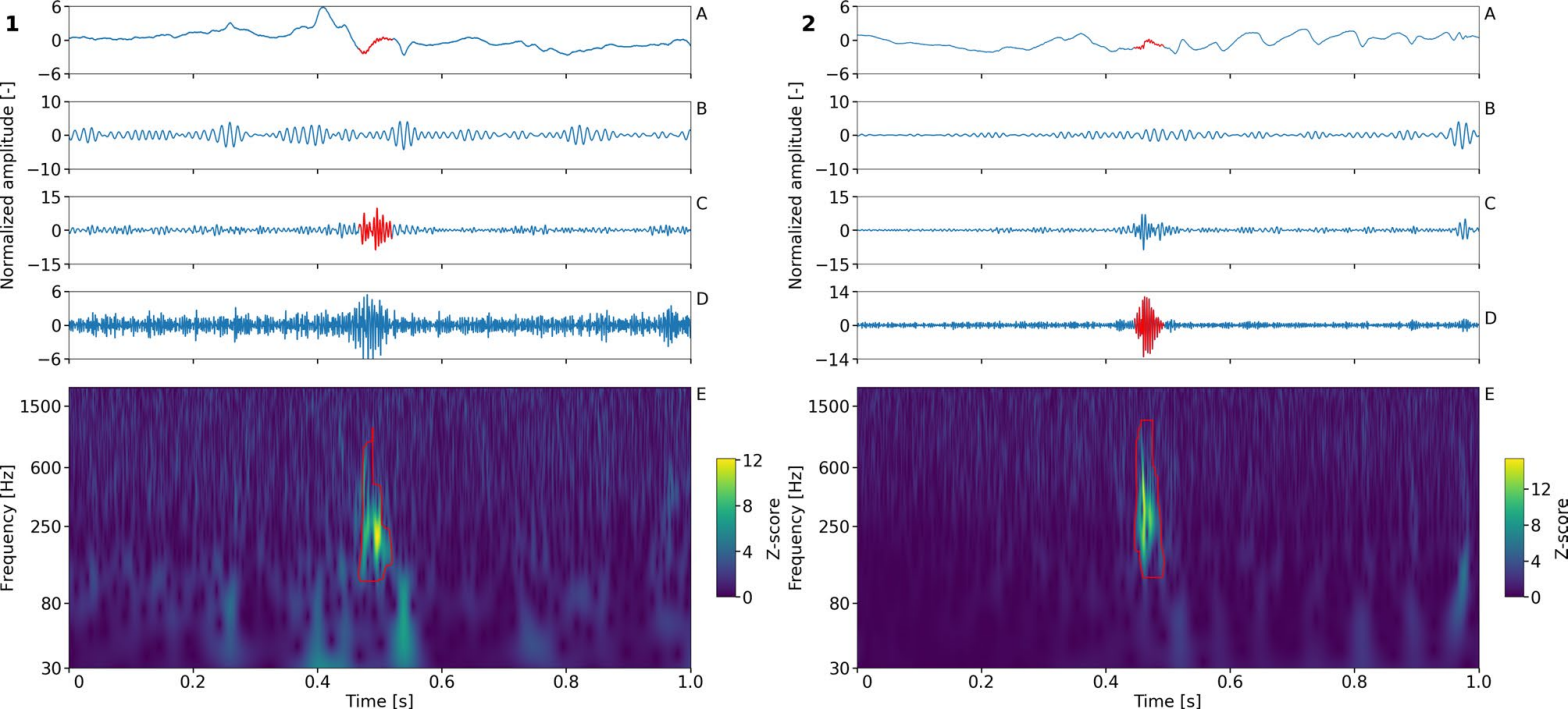
HFO detection. Raw data (A) and band-pass filtered data in the high gamma band frequency (65–80 Hz; (B), ripple band (80–250 Hz; (C) and fast ripple band (250–600 Hz; (D,E) Z-scored power envelopes in a series of log spaced band-pass filtered bands. The time scale of all subplots (A–E) is identical. The detection is represented by red lines in (A,C,E). The brightest spot of the detected event in (E) corresponds to the maximum peak relative amplitude. The frequency of the event is determined by the frequency band in which this peak occurred. The duration of the event is calculated as the difference between earliest onset and latest offset across frequency bands.

The detector of HFOs utilizes a sequence of power envelopes in consecutive logarithmically spaced frequency bands within a 10 s statistical window. The z-score of each separate power envelope is computed and a matrix of the z-scored power envelopes is created. The segment of each power envelope above the threshold (> 3) and with the number of oscillations larger than 1 is marked as a band detection. Band detections overlapping in the temporal domain are joined into one event (Fig. 1).

The relative amplitude is calculated as the highest z-score value of the event and the frequency is determined as the frequency band in which this value occurred. The duration is derived from the first and last value above the threshold across frequency bands. Spectral entropy was computed as the entropy of normalized power spectral density of detected events. Only detections longer than 5 oscillations at the determined frequency were processed.

To investigate how many HFOs occurred simultaneously with spikes and could be influenced by the changes in spike rates, we analyzed the number of HFOs that are superimposed on detected spikes. We analyzed the counts of spike-HFOs as well as standalone HFOs.

### Statistical analysis

The statistical analysis was performed using in-house Python scripts. The individual data sets were first tested for distribution normality with D’Agostino’s normality test. Subsequently, since most of the data sets were not normally distributed, the Wilcoxon rank-sum test was used to investigate differences between studied data sets (the average for individual channels were compared statistically). Bonferroni correction for multiple comparisons was applied where necessary. We also performed an analysis of differences between task-induced and resting HFO rates in each hippocampus (for each contact) using the Wilcoxon paired signed-rank test.

### Ethics approval and consent to participate

We confirm that we have read the Journal’s position on the issues involved in ethical publication and affirm that this report is consistent with those guidelines. All subjects gave their written informed consent prior to the investigation.

## Results

Interictal spikes, ripples, and fast ripples were detected in the hippocampi of all subjects included in the study. The mean percentage of HFOs that occurred simultaneously with spikes was 41% (in detail see Table 2); that is, most HFOs were observed independently of spikes. The degree of success in completing the cognitive task across all patients was at least 96%; this translates to a maximum of 2 errors out of 50 targets.

**Table 2:** Rates of spikes, spike-HFOs and standalone HFOs per 10 min for individuals within resting-state recording.

Subject	Epileptic hippocampus			Non-epileptic hippocampus		
	Spikes	Spike-HFOs	Standalone HFOs	Spikes	Spike-HFOs	Standalone HFOs
1	1950	586	766	205	0	371
2	1623	840	2531	–	–	–
3	4501	2841	838	–	–	–
4	812	477	347	–	–	–
5	1735	1012	299	499	254	241
6	1241	138	988	155	0	347
7	2314	832	706	–	–	–
8	1149	391	1858	–	–	–
9	2767	1450	2204	–	–	–
10	636	295	1168	222	30	576
11	815	277	458	–	–	–
12	–	–	–	308	6	188
13	–	–	–	155	5	376
14	–	–	–	326	168	150
15	3560	1195	1106	–	–	–

**Figure 2:**
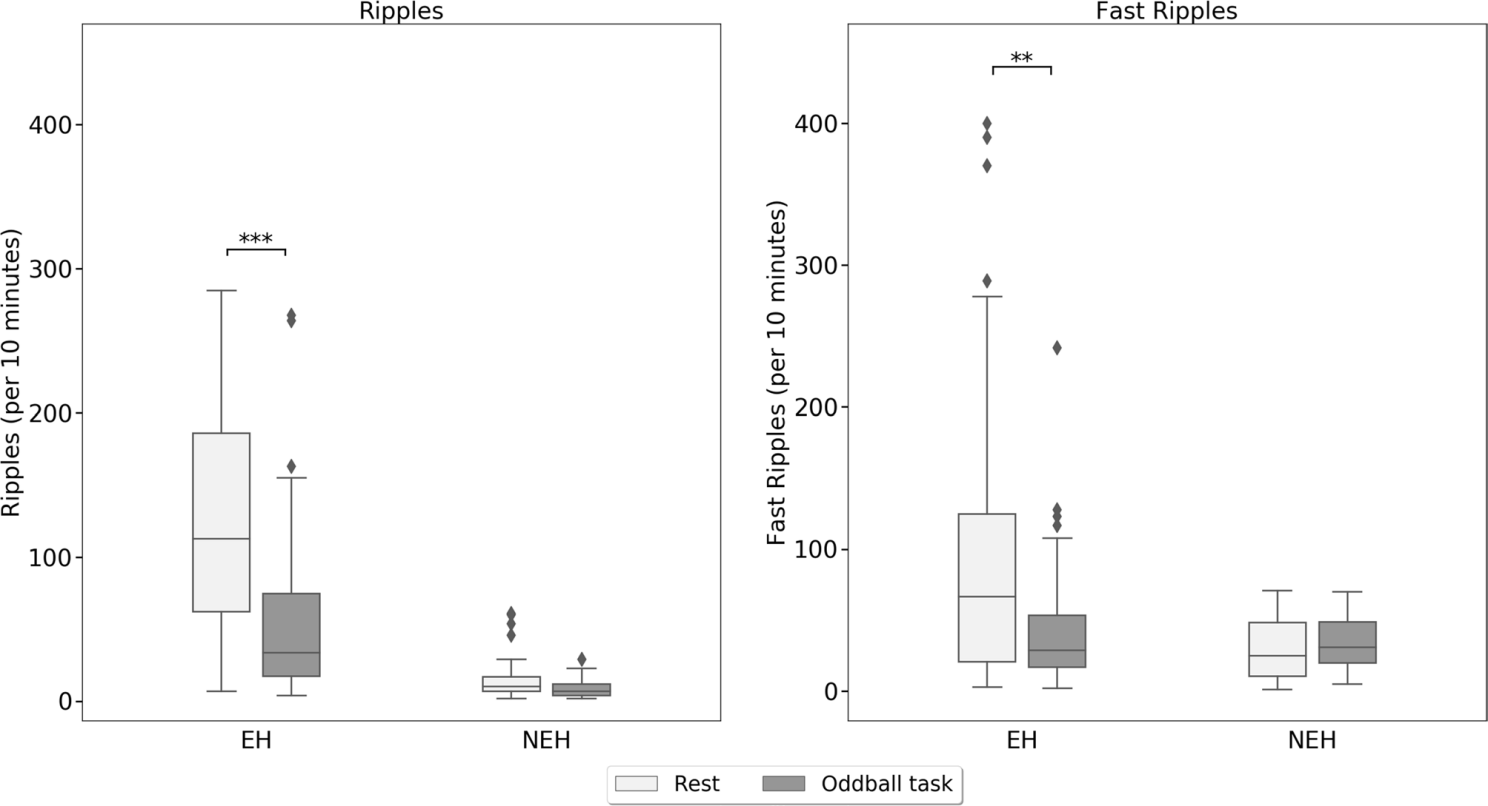
Fast ripple and ripple rates during resting and cognitive-task periods within the epileptic and non-epileptic hippocampi across all investigated subjects. Black asterisks indicate significant differences in epileptic hippocampi (*p* < 0.001). Black diamonds indicate outliers.

**Figure 3:**
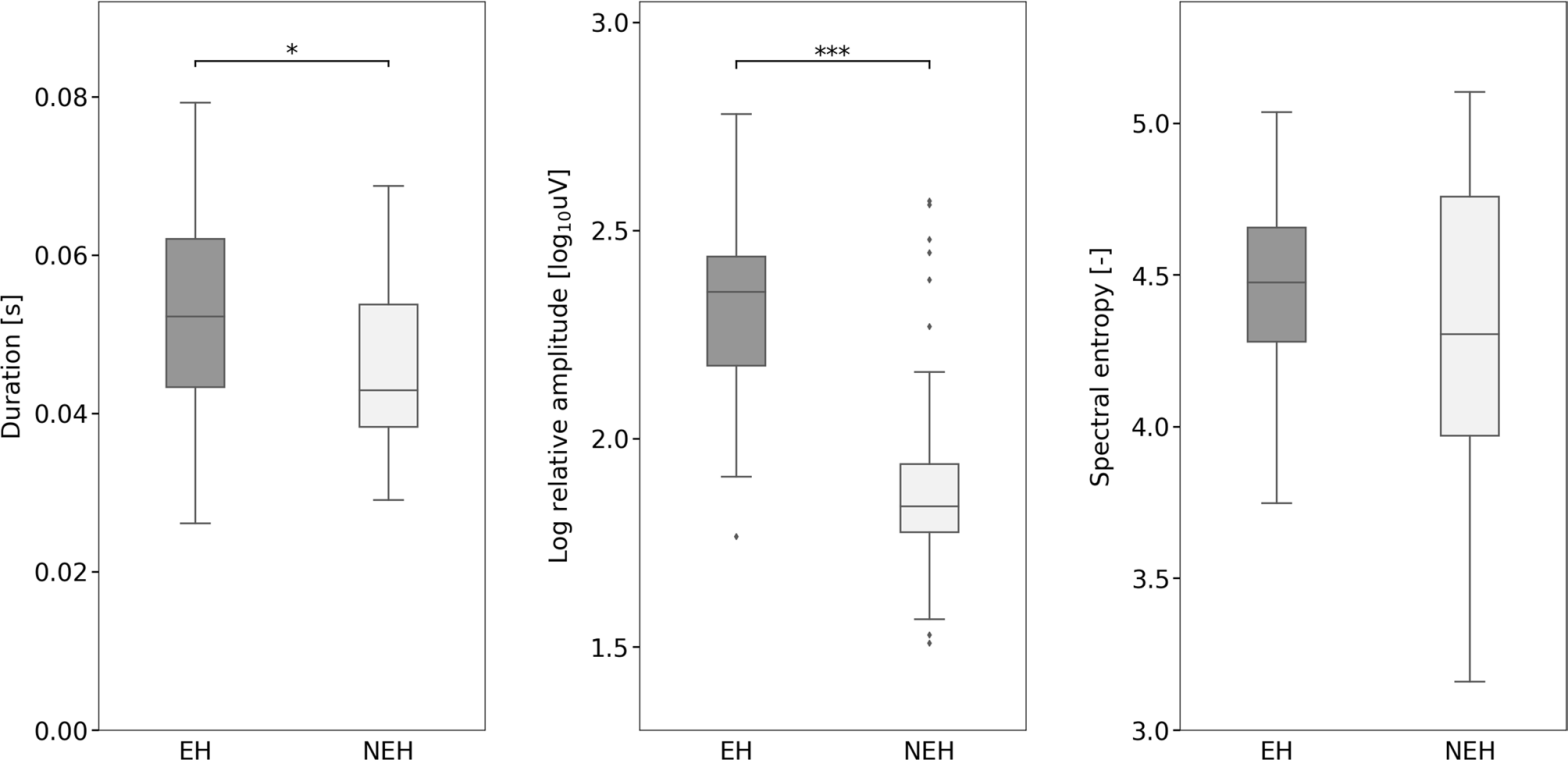
Ripple duration, relative amplitudes and spectral entropy during the resting period within the epileptic and non-epileptic hippocampi across all subjects. Black asterisks indicate significant differences (*p* < 0.001). Black diamonds indicate outliers.

**Figure 4:**
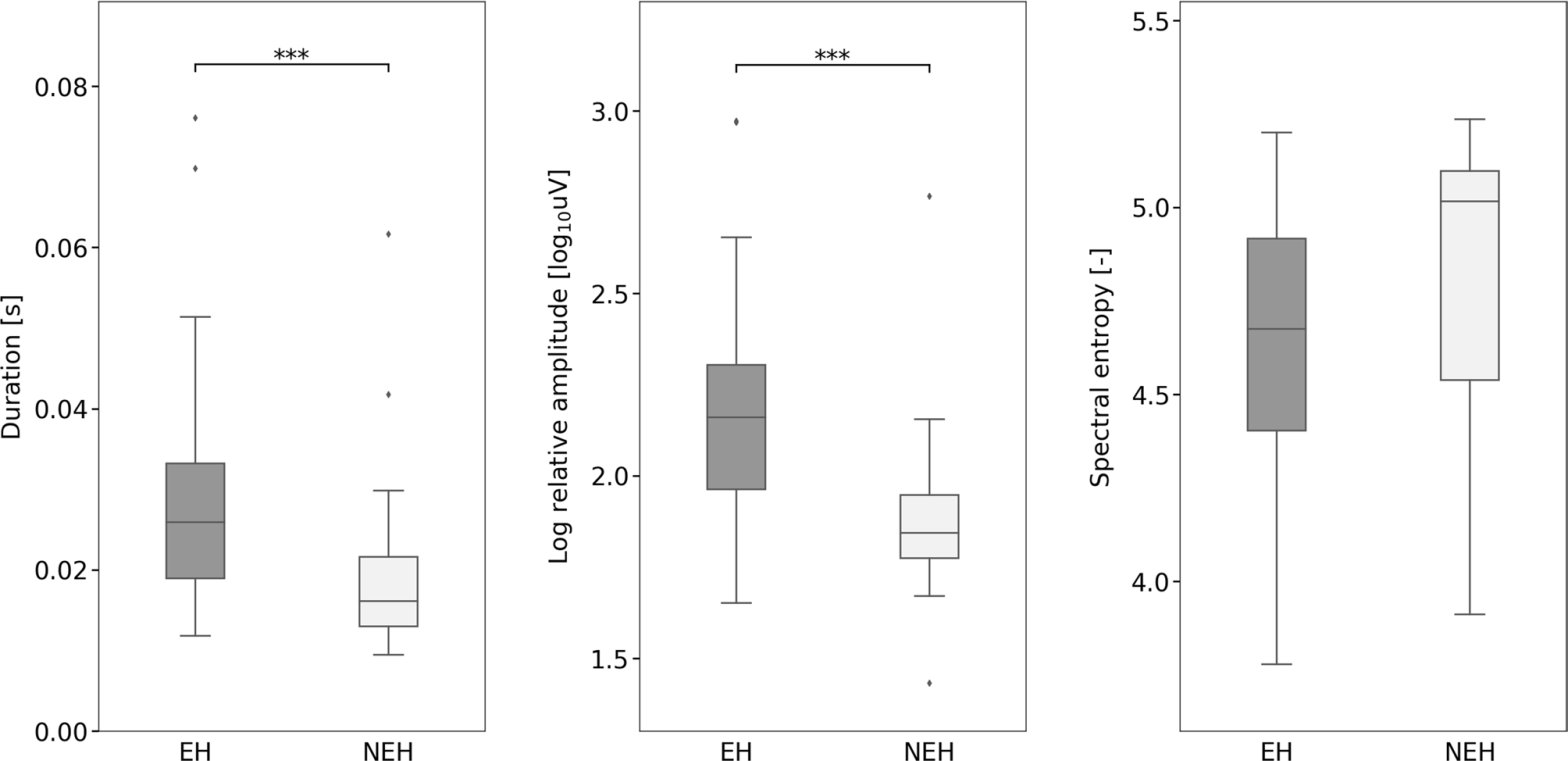
Fast ripple duration, relative amplitudes and spectral entropy during resting periods within the epileptic and non-epileptic hippocampi across all investigated subjects. Black asterisks indicate significant differences (*p* < 0.001). Black diamonds indicate outliers.

During the resting period, a comparison of HFO features revealed that both epileptic and non-epileptic hippocampi exhibited HFOs with similar properties. In the EH compared with NEH, however, we observed both ripples and fast ripples significantly more frequently and with higher relative amplitude and longer duration (Table 3). Furthermore, there was no significant difference in HFO spectral entropy. An illustration of these comparisons for ripples and fast ripples are presented in Figs. 2, 3 and 4.

**Table 3:** HFO characteristics per contact in the ripple and fast ripple ranges during rest and the oddball task.

	Rate (N/10 min)			Log relative amplitude			Duration (ms)			Spectral entropy		
	Rest	Oddball	*p* value	Rest	Oddball	*p* value	Rest	Oddball	*p* value	Rest	Oddball	*p* value
**Ripples**												
NEH	16.06 (± 16.57)	9.0 (± 6.74)	Nonsig	1.92 (± 0.30)	1.95 (± 0.22)	Nonsig	45.73 (± 10.83)	38.39 (± 13.58)	Nonsig	4.32 (± 0.51)	4.71 (± 0.25)	0.001
EH	125.50 (± 75.91)	54.96 (± 52.29)	< 0.001	2.32 (± 0.21)	2.25 (± 0.27)	Nonsig	52.71 (± 10.97)	53.06 (± 14.12)	Nonsig	4.46 (± 0.29)	4.61 (± 0.35)	< 0.05
*p* value	< 0.001	< 0.001		< 0.001	< 0.001		< 0.05	< 0.001		Nonsig	Nonsig	
**Fast ripples**												
NEH	28.52 (± 22.09)	34.54 (± 18.82)	Nonsig	1.89 (± 0.23)	1.88 (± 0.12)	Nonsig	19.14 (± 10.90)	16.40 (± 5.31)	Nonsig	4.82 (± 0.39)	4.89 (± 0.22)	Nonsig
EH	94.38 (± 93.88)	41.26 (± 39.47)	< 0.001	2.16 (± 0.27)	2.02 (± 0.25)	< 0.005	27.51 (± 11.55)	21.75 (± 8.66)	< 0.005	4.65 (± 0.34)	4.87 (± 0.34)	< 0.001
*p* value	< 0.005	Nonsig		< 0.001	< 0.01		< 0.01	< 0.01		Nonsig	Nonsig

The mean HFO rate in the resting period across all contacts was 219.9 ± 151.6 and 40.1 ± 44.3 per 10 min within EH and NEH, respectively. In cognitive-task periods, the mean HFOs rate within EH and NEH changed to 95.5 ± 82.9 and 43.3 ± 19.0 per 10 min, respectively. The change of HFO rate during the cognitive task was significant in EH (*p* < 0.001) but not in NEH. Similar results were revealed by statistical analysis of the differences between the task-induced and the resting period HFO rates using Wilcoxon signed-rank test: A significant reduction of HFO was observed only in contacts within EH (*p* < 0.001).

HFO rates were significantly different in EH compared to NEH during resting state as well as during the cognitive task (*p* < 0.001 and *p* = 0.002, respectively). Looking at HFOs separately in the ripple and fast ripple frequency ranges, we obtain similar results. Ripple and fast ripple rates were significantly reduced during the cognitive task in EH only, however (*p* < 0.001); the rate of HFOs was not significantly influenced by the cognitive task in NEH (Fig. 2).

During the cognitive task, HFOs (both ripples and fast ripples) were detected with the same features as during the resting period in epileptic and non-epileptic hippocampi. However, ripples exhibited higher spectral entropy in both EH and NEH during the cognitive task compared with the resting period. The relative amplitude and duration of R did not change in neither NEH nor EH.

During performance of the cognitive task, more HFOs in the fast ripple frequency range with lower relative amplitude, shorter duration, and higher spectral entropy were detected in EH than during the resting period. In NEH, all characteristics of fast ripples did not differ significantly between rest and the cognitive task. The comparisons of the specific HFO characteristics in the ripple and fast ripple ranges are summarized in Table 3.

Spikes exhibited a similar significant decrease in their rate during the cognitive task in the epileptic hippocampus, but this was observed also in non-epileptic hippocampus. Counts of spike-HFOs as well as standalone HFOs also showed significant reductions during the cognitive task in the epileptic hippocampi (Table 4).

**Table 4:** Spikes, spike-HFOs and standalone HFOs rates per contact during rest and the oddball task.

	Spikes			Spike-HFOs			Standalone HFOs		
	Rate (N/10 min)			Rate (N/10 min)			Rate (N/10 min)		
	Rest	Oddball	*p* value	Rest	Oddball	*p* value	Rest	Oddball	*p* value
NEH	27.15 (± 18.86)	14.13 (± 13.57)	< 0.01	0.72 (± 0.97)	0.88 (± 1.76)	Nonsig	33.88 (± 29.69)	38.88 (± 19.34)	Nonsig
EH	187.9 (± 120.34)	114.00 (± 83.59)	< 0.001	98.68 (± 77.77)	41.25 (± 47.30)	< 0.001	122.51 (± 122.65)	56.88 (± 68.94)	< 0.001
*p* value	< 0.001	< 0.001		< 0.001	< 0.001		< 0.001	Nonsig	

## Discussion

Widespread cortical and subcortical neuronal networks are thought to be coordinated into synchronous oscillations spanning ripples or fast ripples frequency ranges during cognitive phenomena but also pathologic epileptic processes^20^. In this study, we investigated HFOs only in the hippocampus, which plays a pivotal role in both cognitive (especially learning and memory) and epileptogenic processes and which is the most studied brain structure in relation to HFOs. Unsurprisingly, we observed that the ripples and fast ripples were detected at relatively low rates in NEH but at much higher rates in the EH (seizure onset zone), which confirms previously published data concerning the pathogenicity of this phenomena^4,18,39,40^. Importantly, our results not only confirm our previous findings of significantly different behavior of ripples within the EH and NEH, suggesting diverse mechanisms of their generation^8^, but also extend these previous findings by revealing similar results in the fast ripples frequency range.

Our results suggests that a distinction between epileptic and non-epileptic hippocampus cannot be based solely on HFO rates or characteristics at rest; there is no clear limit of HFO rates per 10 min of recording, nor any HFO characteristics that could be used to classify the hippocampal tissue surrounding individual contacts as epileptic or non-epileptic. Only during the very specific discriminative task did we observe a differential decrease in the rate of HFOs in EH. Our results observed within epileptic hippocampi show that its activity is modified by the cognitive task and confirm that a specific discriminative task suppresses pathological HFOs in the epileptic hippocampus, which we discuss below.

Hippocampal and parahippocampal physiological ripples have been proposed to be functionally involved in memory consolidation, strengthening and reorganizing memory traces during both rest and slow wave sleep and providing a link between information transfer and memory formation^20,41–44^. However, this concept of ripples as a physiological phenomenon in the hippocampus during memory-related memory processes has been questioned several times^8,12^. Our previous study showed a significant decrease of ripple rate in epileptic hippocampus during event processing. This may suggest increased involvement of normal hippocampal neurons in physiological cognitive processing and reduced involvement in the epileptic network impelled by synchronously bursting neurons^8^. The prevalence of pathologic ripples is seen usually during non-REM sleep, likely resulting from the sleep-dependent enhancement of network synchronization^5,45–47^. This suggests that a proportion of ripples present in EH are connected to underlying pathological network activity^8^. Our observation that a cognitive task only partly affects general ripple rate within NEH (a large overlap was observed between both EH and NEH) may be explained by the expected physiological role of normal hippocampal neurons during both rest (memory consolidation/awake neuronal replay) and task (complex event discrimination processing) periods^8^.

Conversely, hippocampal fast ripples, VFR, and UFR have been repeatedly reported and considered as biomarkers of epileptogenesis and epileptogenicity, related to pathological processes and occurring in close proximity to the epileptic focus^1,9,10^. VFR and UFR seem to be more localized to the epileptogenic zone than fast ripples; surgical removal of the tissue generating these interictal HFOs leads to favorable surgery outcome^9^. Although fast ripples are considered pathological, they were also detected in a non-epileptic hippocampus^48^. Based on our results, both epileptic and non-epileptic hippocampi have a population of fast ripples with similar properties (i.e. with low fast ripple counts, low relative amplitude, short duration, and non-significant higher spectral entropy), but in the epileptic hippocampus, higher rates of HFOs with extra values of properties tend to occur more often. In other words, the variance is much larger for all the HFO features measured in EH than in NEH.

In line with published data, by evaluating fast ripple occurrence in resting and active periods, we observed the rates of fast ripples spreading to much higher values in EH, and a decreasing number of fast ripples in EH during the discriminative task processing. A similar mechanism like ripple range could be supposed, i.e. the increased involvement of preserved normal hippocampal neurons that are active in some physiological cognitive processing and the reduced involvement of synchronously bursting neurons within the epileptic network generating pathological HFOs^8^. Since the degree of success in completing the cognitive task in all patients was at least 96%, it can be assumed that the changes observed in task performance are really related to the mental processing during the cognitive task. The observed HFO changes in the epileptic hippocampus during the cognitive paradigm could partially reflect the result of activity in many neuronal networks and cognitive processes, including e.g. attention, conscious processing of an event, working memory, stimulus evaluation and response preparation^49,50^. Therefore, the HFO changes observed in the epileptic hippocampus during the oddball task cannot be assigned to a specific function but rather generally related to mental processing.

According to published studies, most brain cortical areas react (with task-induced modulation of high frequency activity) to at least one of the cognitive tasks performed by the patient^51^, except for brain epileptogenic regions that are heavily contaminated by epileptiform activity^51^. Similarly, in a recent animal study, the authors revealed that pathological HFO rate is independent of brain state, though they did not test cognitive load^52^. Based on these results, we would expect that the occurrence of interictal spikes and HFOs is unlikely to be altered during state changes or by stimulation in the epileptic hippocampal region, compared to areas not responsible for seizure generation. Nevertheless, we observed changes just in EH. This would suggest that the epileptic hippocampus was actually participating in processing the stimulus. It is very well known that even within the epileptic hippocampi, some portion of physiological cognitive functions is often preserved. Ewell´s group confirmed that both pathological and non-pathological HFOs can co-occur in the same memory circuits and moreover, up to 28% CA1 principal cells participate in generating both events^52^. However, as can be seen, the majority of hippocampal neurons are modulated by only one event type or the other^52^. Besides clinical and neuropsychological indices, several studies with intracerebral event-related potentials detected cognitive P3 phenomena in both normal and epileptic hippocampi^53–55^. In epileptic hippocampi, these ERPs are often changed but not completely missing.

As mentioned already, in our study fast ripples were also observed in NEH distant from the epileptogenic zone. The fast ripples detected in NEH cannot be clearly defined as pathological or physiological or as a manifestation of the propagation of pathological HFO generated elsewhere. But this propagation effect will not play a significant role, as SEEG measures the local field potentials generated within a centimeter radius and the field formed by neurons over a centimeter from a recording site contribute only a marginal part of the signal^56^.

Moreover, which is essential, the cognitive task obviously did not change the rate of HFOs in NEH. This of course raises the question of whether fast ripples are the result of the non-epileptic activity of neurons in the NEH, since the fast ripple number did not change between resting state and cognitive task. If there were only pathological fast ripples within NEH, we assume that their number would decrease during a cognitive task, similarly to what we found in EH. Not observing a significant change in HFO rate within non-epileptic hippocampi, we hypothesize that healthy hippocampal neurons activated in our specific cognitive task are not involved in physiological HFO genesis. It is widely accepted that physiological HFOs are reflecting memory consolidation and are much more active during sleep^20,41–44^; this is the opposite of our task, which demands a very high attentional load.

Actually, the phenomenon of physiological fast ripples (up to 600 Hz) induced in the hippocampus during a cognitive task was also described in a recent study by Kucewicz et al.^21^. The number of induced HFO was decreasing with increasing frequencies. Most of these induced oscillations lasted between 10 and 25 ms, similarly to the gamma cycle synchronization time frame (correlating with memory formation, loading and maintenance) and the time window for synaptic interactions of neuronal ensembles^21^. This finding supports a physiological origin of fast ripples also in the hippocampus, not only within the primary motor cortex, somatosensory cortex, and visual cortices as was previously published^23,24,26,57^. All things considered, Kucewicz et al.^21^ hypothesized that these induced both ripples and fast ripples likely reflect the coordinated activity of a number of stimulus-specific neurons responding to stimuli. Finally, these phenomena play an important role for fast network synchronization in human cognition.

Concerning the HFO entropy, we found that fast ripples had higher entropy during oddball task than in the rest in the EH. Our results are congruent with those published recently by Liu et al.^58^. Consistently, in all patients, the typical HFOs with the highest degree of waveform similarity (in our case, we can use the term “low entropy”) were seen within epileptogenic tissue only, whereas HFOs embedded in random waveforms (high entropy) were generated by sites in the functional regions independent from the epileptogenic locations^58^. The repetitive waveform pattern was evident in fast ripple range also in our data. This result confirms the possibility of physiological fast ripples in NEH and reduced pathological FR in EH, since these oscillations had higher entropy than those seen during rest in EH and therefore probably have a different origin.

Additionally, we have shown that the “physiological” HFOs have significantly different properties from “pathological” HFOs, primarily shorter duration and lower amplitude^21,24^. In line with this, during the cognitive task in EH the fast ripples were detected with lower mean relative amplitude and shorten duration. In summary, during the cognitive task, more fast ripples were observed in EH with similar characteristics as in the NEH.

The fundamental question remains of whether the resting state and task-related ripples and fast ripples (normal and pathological) exhibit similar or different mechanisms of generation or possess any functional significance. As was shown, HFOs can simply represent a marker of highly activated and synchronized neurons, regardless of the structure or mechanism underlying them. These high frequency signals appear to aggregate local (spiking) activity of neuronal populations or network oscillations^59^. However, the spectral content of local field potential oscillations, which reflects high spectral components arising from sharply contoured transients, is not considered true/standalone HFOs in the invasive EEG^36,60^. Neurons firing broader spikes contaminate the local field potential to a greater extent because their waveforms have stronger components in lower frequencies than short spikes. Moreover, neurons that fire coupled to a certain rhythm and spike synchrony can increase the extent of spike contamination^34^. Signals occurring over larger spatial extents are expected to have greater effect on high frequencies and contribute to a broader range of frequencies^34^. In our study we analyzed true HFOs in the majority of cases as the mean percentage of HFOs that occurred simultaneously with spikes was only 35% (46% in EH and 6% in NEH). We also analyzed the counts of spike-HFOs as well as standalone HFOs. Both analyses showed significant reduction during the cognitive task in the epileptic hippocampus.

Physiological HFOs result from phasic inhibitory input on the soma of pyramidal cells, while epileptic HFOs, usually superimposed on interictal epileptiform sharp waves, appear to reflect the field potentials which are formed by the activity from clusters of abnormal synchronously bursting pyramidal cells, generating population spikes, and decreased inhibitory interneuron firing^6,15^. Based on the functioning of synaptic transmission, the contribution of this mechanism to HFO genesis is limited to approximately 80–150 Hz^61^. The true high frequency local field potential oscillations above ~ 250 Hz are above the physiological firing rate of pyramidal neurons and cannot be generated by synaptic currents^61^. It is assumed, these local field potential oscillations are an arising rhythm generated by the in and out of phase action potential firing of populations of neuronal cell assemblies or clusters^9,61^. Originally suggested pathologically interconnected neurons emitting hypersynchronous bursts, as the source of fast ripple oscillations^15^ and a population of these clusters might underlie generation of activity above ~ 500 Hz^9^.

The fact that our study consists of analyses of chronic epileptic patients is an obvious limitation. The brain tissue from which the signal is acquired is not organized in the same way as normal tissue; this may lead to a bad and misleading model of physiological human neural processing and functional organization^56^. The possibility of the disease-related processes interfering with the reported physiological oscillations cannot be completely ruled out and must be taken into account, although usually many epileptic patients perform behaviorally as well as normal subjects^56^. Certain caution must be taken when interpreting “normal” results onto normal hippocampal behavior.

It is important to highlight that the majority of previous studies evaluated results drawn from HFO analysis at a group level, and when considering individual patients the rates of HFOs are often highly variable and less specific for epileptic brain localization^12,36^. HFOs could reflect increased cortical excitability, perhaps more than epileptogenicity. Our results support a possible physiological origin of fast ripples as well. Thus, in individual patients, the count of fast ripples may include fast ripples of physiological origin and therefore fast ripples may not be a sensitive and unique biomarker of epileptogenicity^12^. This finding, however, does not alter the fact that pathological fast ripples clearly prevail in epileptic hippocampi.

## Conclusion

Based on our results using a visual oddball task, it is possible to differentiate between epileptic and non-epileptic hippocampi, even though both hippocampi have HFOs with similar features that probably reflect non-pathological phenomena. And so, fast ripples recorded in the hippocampus should not be considered as only a pathological. Our results confirm the distinct impact of a very specific discriminative task processing on ripples and fast ripples within epileptic and non-epileptic hippocampi, particularly the suppression of pathological HFOs in epileptic hippocampus.

## References

[1] Bragin A, Engel J, Wilson CL, Fried I, Buzsáki G (1999). High-frequency oscillations in human brain. Hippocampus.

[2] Worrell GA (2004). High-frequency oscillations and seizure generation in neocortical epilepsy. Brain.

[3] Worrell G, Gotman J (2011). High-frequency oscillations and other electrophysiological biomarkers of epilepsy: clinical studies. Biomark. Med..

[4] Urrestarazu E, Chander R, Dubeau F, Gotman J (2007). Interictal high-frequency oscillations (100–500 Hz) in the intracerebral EEG of epileptic patients. Brain.

[5] Bagshaw AP, Jacobs J, LeVan P, Dubeau F, Gotman J (2009). Effect of sleep stage on interictal high-frequency oscillations recorded from depth macroelectrodes in patients with focal epilepsy. Epilepsia.

[6] Engel J, Bragin A, Staba R, Mody I (2009). High-frequency oscillations: What is normal and what is not?. Epilepsia.

[7] Brázdil M (2010). Interictal high-frequency oscillations indicate seizure onset zone in patients with focal cortical dysplasia. Epilepsy Res..

[8] Brázdil M (2015). Impact of cognitive stimulation on ripples within human epileptic and non-epileptic hippocampus. BMC Neurosci..

[9] Brázdil M (2017). Very high-frequency oscillations: Novel biomarkers of the epileptogenic zone. Ann. Neurol..

[10] Jacobs J (2012). High-frequency oscillations (HFOs) in clinical epilepsy. Prog. Neurobiol..

[11] Usui N (2015). Significance of very-high-frequency oscillations (over 1,000 Hz) in epilepsy. Ann. Neurol..

[12] Roehri N (2018). High-frequency oscillations are not better biomarkers of epileptogenic tissues than spikes. Ann. Neurol..

[13] Engel J, da Silva FL (2012). High-frequency oscillations—Where we are and where we need to go. Prog. Neurobiol..

[14] Buzsáki G, Horváth Z, Urioste R, Hetke J, Wise K (1992). High-frequency network oscillation in the hippocampus. Science.

[15] Bragin A, Benassi SK, Kheiri F, Engel J (2011). Further evidence that pathologic high-frequency oscillations are bursts of population spikes derived from recordings of identified cells in dentate gyrus. Epilepsia.

[16] Jiruska P (2013). Synchronization and desynchronization in epilepsy: Controversies and hypotheses. J. Physiol..

[17] Jiruska P (2017). Update on the mechanisms and roles of high-frequency oscillations in seizures and epileptic disorders. Epilepsia.

[18] Jacobs J (2008). Interictal high-frequency oscillations (80–500 Hz) are an indicator of seizure onset areas independent of spikes in the human epileptic brain. Epilepsia.

[19] Bartolomei F (2016). What is the concor-dance between the seizure onset zone and the irritative zone? A SEEG quantified study. Clin. Neurophysiol..

[20] Buzsáki G, Silva FL (2012). High frequency oscillations in the intact brain. Prog. Neurobiol..

[21] Kucewicz MT (2014). High frequency oscillations are associated with cognitive processing in human recognition memory. Brain.

[22] Alkawadri R (2014). The spatial and signal characteristics of physiologic high frequency oscillations. Epilepsia.

[23] Nagasawa T (2012). Spontaneous and visually driven high-frequency oscillations in the occipital cortex: Intracranial recording in epileptic patients. Hum. Brain Mapp..

[24] Matsumoto A (2013). Pathological and physiological high-frequency oscillations in focal human epilepsy. J. Neurophysiol..

[25] Pail M (2017). Frequency-independent characteristics of high-frequency oscillations in epileptic and non-epileptic regions. Clin. Neurophysiol..

[26] Wang S (2013). Ripple classification helps to localize the seizure-onset zone in neocortical epilepsy. Epilepsia.

[27] Zijlmans M (2017). How to record high-frequency oscillations in epilepsy: A practical guideline. Epilepsia.

[28] Jiruska P, Bragin A (2011). High-frequency activity in experimental and clinical epileptic foci. Epilepsy Res..

[29] Crépon B (2010). Mapping interictal oscillations greater than 200 Hz recorded with intracranial macroelectrodes in human epilepsy. Brain.

[30] von Ellenrieder N, Frauscher B, Dubeau F, Gotman J (2016). Interaction with slow waves during sleep improves discrimination of physiologic and pathologic high-frequency oscillations (80–500 Hz). Epilepsia.

[31] Bruder JC (2017). Physiological ripples associated with sleep spindles differ in waveform morphology from epileptic ripples. Int. J. Neural. Syst..

[32] Sakuraba R (2016). High frequency oscillations are less frequent but more specific to epileptogenicity during rapid eye movement sleep. Clin. Neurophysiol..

[33] Malinowska U, Bergey GK, Harezlak J, Jouny CC (2015). Identification of seizure onset zone and preictal state based on characteristics of high frequency oscillations. Clin. Neurophysiol..

[34] Waldert S, Lemon RN, Kraskov A (2013). Influence of spiking activity on cortical local filed potentials. J. Physiol..

[35] Kucewicz MT (2017). Dissecting gamma frequency activity during human memory processing. Brain.

[36] Cimbalnik J, Kucewicz MT, Worrell G (2016). Interictal high-frequency oscillations in focal human epilepsy. Curr. Opin. Neurol..

[37] Talairach J (1967). Atlas d´anatomie stéréotaxique du telencéphale: etudes anatomo-radiologiques.

[38] Barkmeier DT (2012). High inter-reviewer variability of spike detection on intracranial EEG addressed by an automated multi-channel algorithm. Clin. Neurophysiol..

[39] Jacobs J (2009). High frequency oscillations in intracranial EEGs mark epileptogenicity rather than lesion type. Brain.

[40] Andrade-Valença L (2012). Interictal high frequency oscillations (HFOs) in patients with focal epilepsy and normal MRI. Clin. Neurophysiol..

[41] Axmacher N, Elger CE, Fell J (2008). Ripples in the medial temporal lobe are relevant for human memory consolidation. Brain.

[42] Carr MF, Jadhav SP, Frank LM (2011). Hippocampal replay in the awake state: A potential substrate for memory consolidation and retrieval. Nat. Neurosci..

[43] Girardeau G, Benchenane K, Wiener SI, Buzsáki G, Zugaro MB (2009). Selective suppression of hippocampal ripples impairs spatial memory. Nat. Neurosci..

[44] Lachaux JP, Axmacher N, Mormann F, Halgren E, Crone NE (2012). High-frequency neural activity and human cognition: Past, present and possible future of intracranial EEG research. Prog. Neurobiol..

[45] Steriade M, Contreras D, Amzica F (1994). Synchronized sleep oscillations and their paroxysmal developments. Trends Neurosci..

[46] Dinner DS (2002). Effect of sleep on epilepsy. J. Clin. Neurophysiol..

[47] de Guzman PH, Nazer F, Dickson CT (2010). Short-duration epileptic discharges show a distinct phase preference during ongoing hippocampal slow oscillations. J. Neurophysiol..

[48] Staba RJ, Wilson CL, Bragin A, Fried I, Engel J (2002). Quantitative analysis of high-frequency oscillations (80–500 Hz) recorded in human epileptic hippocampus and entorhinal cortex. J. Neurophysiol..

[49] Polich J (2007). Updating p300: An integrative theory of P3a and P3b. Clin. Neurophysiol..

[50] Pail M (2016). Connectivity of superior temporal sulcus during target detection. J. Psychophysiol..

[51] Lachaux JP, Lhatoo SD, Kahane P, Lüders HO (2019). Dynamic spectral imaging: Online and offline functional brain mapping using high-frequency activity [50–150 Hz] in SEEG. Invasive Studies of the Human Epileptic Brain.

[52] Ewell LA, Fischer KB, Leibold C, Leutgeb S, Leutgeb JK (2019). The impact of pathological high-frequency oscillations on hippocampal network activity in rats with chronic epilepsy. eLife.

[53] Meador KJ (1987). Limbic evoked potentials predict site of epileptic focus. Neurology.

[54] Puce A, Kalnins RM, Berkovic SF, Donnan GA, Bladin PF (1989). Limbic P3 potentials, seizure localization, and surgical pathology in temporal lobe epilepsy. Ann. Neurol..

[55] Brázdil M (1999). Hippocampal visual P3 potential in lateralization of primary epileptogenic focus and in assessment of hippocampal memory function in temporal lobe epilepsy. Epilepsia.

[56] Lachaux JP, Rudrauf D, Kahane P (2003). Intracranial EEG and human brain mapping. J. Physiol. Paris.

[57] Curio G (2000). Linking 600-Hz “spikelike” EEG/MEG wavelets (“ς-bursts”) to cellular substrates: Concepts and caveats. J. Clin. Neurophysiol..

[58] Liu S (2018). Stereotyped high-frequency oscillations discriminate seizure onset zones and critical functional cortex in focal epilepsy. Brain.

[59] Rich EL, Wallis JD (2017). Spatiotemporal dynamics of information encoding revealed in orbitofrontal high-gamma. Nat. Commun..

[60] Bénar CG, Chauvière L, Bartolomei F, Wendling F (2010). Pitfalls of high-pass filtering for detecting epileptic oscillations: A technical note on “false” ripples. Clin. Neurophysiol..

[61] Menendez de la Prida L, Staba RJ, Dian JA (2015). Conundrums of high-frequency oscillations (80–800 Hz) in the epileptic brain. J. Clin. Neurophysiol..

